# Complex trait relationships between leaves and absorptive roots: Coordination in tissue N concentration but divergence in morphology

**DOI:** 10.1002/ece3.2895

**Published:** 2017-03-19

**Authors:** Ruili Wang, Qiufeng Wang, Ning Zhao, Guirui Yu, Nianpeng He

**Affiliations:** ^1^ College of ForestryNorthwest A&F UniversityYanglingShaanxiChina; ^2^ Synthesis Research Center of Chinese Ecosystem Research NetworkKey Laboratory of Ecosystem Network Observation and ModelingInstitute of Geographic Sciences and Natural Resources ResearchChinese Academy of SciencesBeijingChina; ^3^ Laboratory of Remote Sensing and Geospatial ScienceCold and Arid Regions Environmental and Engineering Research InstituteChinese Academy of SciencesLanzhouGansuChina

**Keywords:** above‐ and belowground linkage, absorptive roots, leaves, morphological trait, N concentration

## Abstract

Leaves and absorptive roots (i.e., first‐order root) are above‐ and belowground plant organs related to resource acquisition; however, it is controversy over whether these two sets of functional traits vary in a coordinated manner. Here, we examined the relationships between analogous above‐ and belowground traits, including chemical (tissue C and N concentrations) and morphological traits (thickness and diameter, specific leaf area and root length, and tissue density) of 154 species sampling from eight subtropical and temperate forests. Our results showed that N concentrations of leaves and absorptive roots were positively correlated independent of phylogeny and plant growth forms, whereas morphological traits between above‐ and belowground organs varied independently. These results indicate that, different from plant economics spectrum theory, there is a complex integration of diverse adaptive strategies of plant species to above‐ and belowground environments, with convergent adaptation in nutrient traits but divergence in morphological traits across plant organs. Our results offer a new perspective for understanding the resource capture strategies of plants in adaptation to heterogeneous environments, and stress the importance of phylogenetic consideration in the discussion of cross‐species trait relationships.

## 1 Introduction

Plant traits could illustrate the fundamental trade‐offs that affect the fitness and success of individual plants in a given environment (Reich, 2014; Violle et al., 2007). Among various plant traits, variation in morphological traits could directly reflect the resource acquisition strategies of plants and their adaptation to external environments (Li et al., 2015; Poorter, Niinemets, Poorter, Wright, & Villar, 2009; Reich, 2014); chemical traits (e.g., tissue N concentration) are often related to these process in metabolic activities and thus play an important role in controlling carbon assimilation and primary production (Kerkhoff, Fagan, Elser, & Enquist, 2006; Kong et al., 2016; Wright et al., 2004). More importantly, both morphological and chemical parameters are relatively easy to measure and thus can be used effectively in the trait comparative studies across a broad of species, especially at the large scale (e.g., Chen, Zeng, Eissenstat, & Guo, 2013; Kong et al., 2014; Wright et al., 2004). However, compared with leaf traits, far less is known about root system traits, and it is unclear whether absorptive root (i.e., first‐order root) traits covary with the corresponding foliar traits (Bardgett, Mommer, & De Vries, 2014; Weemstra et al., 2016).

A commonly proposed theory is that, under strong environmental selection and biophysical constraints, there is coordination among above‐ and belowground traits, tissue biomass construction costs, and uptake of resources (“plant economics spectrum theory”; Freschet, Cornelissen, van Logtestijn, & Aerts, 2010; Reich, 2014). Some stoichiometric studies have shown that the leaf and root N (Craine, Lee, Bond, Williams, & Johnson, 2005; Liu et al., 2010; Valverde‐Barrantes, Smemo, & Blackwood, 2015) and P concentrations (Geng, Wang, Jin, Liu, & He, 2014; Holdaway, Richardson, Dickie, Peltzer, & Coomes, 2011; Kerkhoff et al., 2006) are positively correlated, providing support for the nutrient portion of the plant economics spectrum hypothesis. However, among morphological traits, results of trait relationships are mixed and dependent on spatial scale, geographical region, and plant growth form (Craine et al., 2005; Geng et al., 2014; Holdaway et al., 2011). For example, the reported specific leaf area–specific root length (SLA–SRL) relationship varied from positive (Liu et al., 2010; Withington, Reich, Oleksyn, & Eissenstat, 2006) to negative (Li & Bao, 2015), nonsignificant (Chen et al., 2013; Tjoelker, Craine, Wedin, Reich, & Tilman, 2005; Valverde‐Barrantes et al., 2015), or an environment‐dependent relationship (Geng et al., 2014; Holdaway et al., 2011). More generally, tissue density and organ thickness were found to be poorly or not correlated between leaves and roots (Craine et al., 2005; Holdaway et al., 2011; Kembel & Cahill, 2011). Thus, such different relationships in terms of morphology suggest that the trait correlations between above‐ and belowground resource‐acquiring organs deserve further attention.

This study aims to determine whether the general relationships between corresponding leaf and absorptive root traits existed by using a wide range of species taxa and biomes. We hypothesize that there should be complex trait relationships between leaves and absorptive roots. Specifically, positive correlations in N concentrations of leaves and absorptive roots are expected, because root‐acquired resources from soil, especially N and P, are eventually transferred to the leaves for overall metabolic activity and plant growth (Mommer & Weemstra, 2012; Westoby & Wright, 2006). On the other hand, morphological parameters have been found to show greater cross‐species variation and higher phylogenetic conservatism than chemical parameters for both absorptive roots (Kong et al., 2014; Valverde‐Barrantes et al., 2015) and leaves (Li et al., 2015). Such strong phylogenetic effect may lead to that plant traits are more similar among closely related species than those distantly related (Blomberg, Garland, & Ives, 2003; Münkemüller et al., 2012; Paradis, 2012). Thus, we predict that relationships between leaf and absorptive root morphological traits may depend on plant phylogeny, and this possible coordination would disappear after adjusting for phylogenetic relatedness. Also, the possible morphological linkage between above‐ and belowground organs may differ between woody and nonwoody plant species because of the difference in their root branch systems (Geng et al., 2014; Roumet et al., 2016).

To test these hypotheses, we sampled leaf and absorptive root traits of 154 species from eight subtropical and temperate forests in eastern China, and measured the analogous leaf and root traits related to plant resource acquisitive strategies. Pairs of morphological traits were leaf thickness (LT) and root diameter (RD); SLA and SRL; and tissue density (LTD and RTD); chemical traits included leaf and root C (LC and RC) and N (LN and RN) concentrations. A species‐level phylogenetic tree for all species was generated to determine whether trait correlation is caused by selection pressure or constraint by phylogenetic relatedness among species (Blomberg & Garland, 2002; Kembel & Cahill, 2011; Münkemüller et al., 2012). We also divided all species into woody and nonwoody species to investigate how the trait relationships vary between two growth forms.

## 2 Materials and Methods

### 2.1 Study site

Eight natural forests along the North‐South Transect of Eastern China (NSTEC)—Dinghu Mountain, Jiulian Mountain, Shennongjia, Taiyue Mountain, Dongling Mountain, Changbai Mountain, Liangshui, and Huzhong—were selected to conduct field sampling (Table S1). These ecosystems span latitudes from 23 to 51°N, with a mean annual temperature ranging from −4.4 to 20.9°C and a mean annual precipitation from 481.6 to 1,927 mm. Soils also vary markedly: Soil type ranges from subtropical red soils in the southern sites to brown soils in the northern sites; total soil N ranges from 1.76 to 6.37 mg/g and total soil P from 203.68 to 1797.88 mg/kg. Correspondingly, vegetation type varies from subtropical evergreen forest to temperate deciduous forest and cold‐temperate coniferous forest.

### 2.2 Sampling and trait measurement

During July and August of 2013, samples of leaves and absorptive roots were collected from eight forest sites. At each site, four experimental plots (30 × 40 m) were set up, and floristic and environmental surveys were performed (see Wang et al., 2015 for details). In each plot, the leaves and roots of dominant plant species in each layer were collected according to a unified protocol. For leaf sampling, 20 mature and undamaged leaves (sun leaves were selected for woody species) were collected from four individuals of each plant species. Leaf traits, including SLA (m^2^/kg) and LT (mm), were measured following the procedure described in Cornelissen et al. (2003). LTD (g/cm^3^) was calculated as the inverse of SLA divided by LT. Total leaf C and N concentrations (mg/g) were determined by dry combustion using an elemental analyzer (Vario MAX CN Elemental Analyzer, Elementar, Germany).

For most species, we chose the same individual for leaves and roots samples. Root samples were collected according to the procedure described in Guo et al. (2008). For each woody species, root sampling in undisturbed soil involved excavating the main root stem from the surface soil (0–20 cm) near the plant basal stem, then tracing the intact root system to the lateral root clusters. Root clusters with intact branch orders were cut from the main lateral woody roots and then immediately transported in the laboratory for further morphological and chemical analyses. The whole root systems of nonwoody species were obtained by using a pick or shovel.

In the laboratory, after careful cleaning of adhering soil particles and organic matter, the root branching clusters were kept moist with deionized water and dissected into different branch orders by hand, following Pregitzer et al. (2002). Here, we focused only on the most distal roots (i.e., the first‐order roots) and defined them as absorptive roots, as only the most distal first‐order roots with the most rapid turnover and highest metabolic activity are functionally comparable to leaves as resource acquisition organs (Comas & Eissenstat, 2009; Guo et al., 2008; Kong et al., 2014). Also, to ensure a fair comparison between plant types, the first‐order root was considered both for woody and nonwoody species. These root samples were used to measure five key belowground traits in absorptive roots: RD (mm), SRL (m/g), RTD (g/cm^3^), and tissue C and N concentrations. The root diameter, length, and volume data were obtained by analyzing the scanned root samples with WinRHIZO 2009 (Regent Instruments, Canada). The RTD was calculated as root dry mass divided by root volume. Owing to the limited amount of absorptive roots, root C and N concentrations (mg/g) were determined using an isotope ratio mass spectrometer (MAT253, Thermo Electron Corporation, Germany).

### 2.3 Species and phylogeny

A total of 310 species‐at‐site observations including a set of root and leaf samples were taken from eight forest ecosystems, representing 154 plant species in 116 genera, 61 families, and 28 orders. These species covered a broad phylogenetic range with 130 angiosperms, 11 gymnosperms, and 13 pteridophytes. The online software Phylomatic version 3 (http://www.phylodiversity.net/phylomatic) (Webb, Ackerly, & Kembel, 2008) was used to build a species‐level phylogenetic tree based on the megatree R20120829 (Figure S1). Age estimates for nodes in the tree were taken from Wikström, Savolainen, and Chase (2001), and branch lengths were adjusted using the “bladj” function in the software Phylocom (Webb et al., 2008). Because phylogenetic analyses were calculated based on the full resolved tree structure, we resolved the polytomies by arbitrarily transforming all multichotomies into a series of dichotomies with zero‐length branches (function “multi2di” in R package “ape”) (Münkemüller et al., 2012; Webb et al., 2008).

### 2.4 Statistical analysis

Due to the skewed distributions of data (Figure S2), leaf and absorptive root traits were log_10_‐transformed when it was necessary to obtain approximate normality and homogeneity of residuals. Species‐by‐site data were averaged for each species, and the average for each species was then classified into woody and nonwoody species. A one‐way analysis of variance (ANOVA) was used to compare leaf and absorptive root traits between woody and nonwoody species.

To assess the phylogenetic conservatism between above‐ and belowground traits, we calculated phylogenetic signal in all traits by performing the Blomberg's *K* statistic (Blomberg et al., 2003) and Pagel's λ tests (Pagel, 1999). Both indies reflect the phylogenetic dependence of observed trait data with respect to a pure Brownian model of evolution, but Blomberg's *K* was suitable to capture the effects of changing evolutionary rates in simulation experiments, while Pagel's λ performed better for discriminating between complex models of trait evolution (Münkemüller et al., 2012). Blomberg's *K* values vary from 0 (no signal) to infinity and Pagel's λ from 0 to 1. A larger value in both methods indicates a greater phylogenetic conservatism for the given trait. Significance was testing via comparison of the variance of standardized contrasts to random values obtained by shuffling trait data across the tips of the tree 999 times. Both tests were performed using the “phylosig” procedure in the R package “phytools.”

To evaluate the impacts of phylogenetic autocorrelation on the trait relationships between leaf and absorptive roots, we compared ordinary least squares (OLS) regressions with phylogenetic generalized least squares (PGLS) analyses using all species data and two subsets of woody and nonwoody species, respectively. The latter method accounts for evolutionary association among species and yields unbiased regression coefficients and significance levels (Paradis, 2012; Revell, 2010). For phylogenetic analyses, we first assessed different phylogenetic correlation structures (Brownian, Martin's, and Pagel's) prior to any further investigations of trait relationships and selected the best method to take the phylogeny into account by comparing Akaike's information criterion (AIC) for these models. These preliminary analyses showed that Pagel's λ was the best phylogenetic correlation structure (lowest AIC, Table S2). We thus applied this methodology in all tests of the trait relationships in our study, in which phylogenetic regression was performed with a phylogenetic tree whose internal branches were multiplied by λ, leaving the tip branches at their original length (Paradis, 2012; Revell, 2010). Here, λ was estimated with maximum likelihood using the “gls” function from the R package “nlme.”

Lastly, a phylogenetic principal component analysis (pPCA) was performed with the whole set of plant traits (10 traits) for all 154 species to test whether the trait syndrome of absorptive roots is comparable to that of leaves. Then we performed pPCA for each subset for woody and nonwoody species, separately. Different from the ordinary principal components analysis (PCA), pPCA is a method recently proposed for dealing multivariate data in a way that takes into account the phylogenetic nonindependence among species means (Revell, 2009). In this study, both results of ordinary and phylogenetic PCA were presented as we aimed to show that correcting for the phylogenetic impacts on above‐ and belowground trait relationships. These analyses were carried out using log_10_‐transformed species means with the R packages “phytools” and “stats.”

All statistical analyses were performed in the R 3.3.1 statistical platform (R Core Development Team, http://www.r-project.org/).

## 3 Results

### 3.1 Variation in leaf and absorptive root traits

Across the 154 species, there was ninefold variation in first‐order root diameter, ranging from a minimum of 0.09 mm in *Cardamine leucantha* to a maximum of 0.83 mm in *Sarcosperma laurinum* (Figure S2), with an overall coefficient of variation (CV) of 0.33. In general, leaf and absorptive root traits had greater variation in morphological traits than in chemical traits (Table 1 and Figure S2). SRL, LT, and SLA had the greatest proportional variation among observations (61, 42, and 28 orders of magnitude, respectively). These variations were far larger than those in tissue C and N concentrations (two–sevenfold) of both leaves and absorptive roots.

**Table 1:** Summary statistics of leaf and absorptive root traits between woody and nonwoody species

	Trait	Woody species (*n *=* *112)	Nonwoody species (*n *=* *42)	All species (*n *=* *154)
Leaf	LT (mm)	0.18 ± 0.11a	0.13 ± 0.06b	0.17 ± 0.11
	SLA (m^2^/kg)	15.62 ± 8.84a	25.39 ± 12.48b	18.23 ± 10.82
	LTD (g/cm^3^)	0.52 ± 0.19a	0.42 ± 0.20b	0.49 ± 0.20
	LC (mg/g)	473.56 ± 28.19a	434.44 ± 31.20b	463.09 ± 33.77
	LN (mg/g)	22.62 ± 7.46a	26.32 ± 7.71b	23.62 ± 7.69
Absorptive root	RD (mm)	0.28 ± 0.12a	0.25 ± 0.09a	0.27 ± 0.11
	SRL (m/g)	117.63 ± 87.93a	172.86 ± 96.76b	132.45 ± 93.49
	RTD (g/cm^3^)	0.23 ± 0.07a	0.18 ± 0.07b	0.21 ± 0.07
	RC (mg/g)	522.61 ± 68.96a	503.98 ± 75.89a	517.40 ± 71.29
	RN (mg/g)	19.92 ± 5.54a	19.27 ± 6.42a	19.74 ± 5.80

*n*, species number; LT, leaf thickness; SLA, specific leaf area; LTD, leaf tissue density; LC, leaf carbon concentration; LN, leaf nitrogen concentration; RD, root diameter; SRL, specific root length; RTD, root tissue density; RC, root carbon concentration; RN, root nitrogen concentration.

Values are mean ± 1 *SD*. In each row, different letters indicate significant differences between woody and nonwoody species (*P *<* *.05).

Above‐ and belowground traits also differed greatly between woody and nonwoody species (Table 1). Woody species exhibited higher LT and tissue density in contrast to nonwoody plants (LT: *F*_1,152_ = 5.106, *P *=* *.025; LTD: *F*_1,152_ = 20.65, *P *<* *.001; RTD: *F*_1,152_ = 31.53, *P *<* *.001), while nonwoody species had relatively higher SLA and SRL (SLA: *F*_1,152_ = 25.22, *P *<* *.001; SRL: *F*_1,152_ = 18.65, *P *<* *.001). However, RD did not show significant difference between two plant growth types (*F*_1,152_ = 3.10, *P *=* *.080). For chemical traits, nonwoody species had significantly lower LC but higher LN when compared with their woody counterparts (LC: *F*_1,152_ = 16.36, *P *<* *.001; LN: *F*_1,152_ = 15.42, *P *<* *.001), but no significant differences were observed in RC and RN between woody and nonwoody plants (RC: *F*_1,152_ = 1.13, *P *=* *.291; RN: *F*_1,152_ = 0.04, *P *=* *.836).

### 3.2 Phylogenetic effect on leaf and absorptive root traits

When data of all species were pooled together, most of the ten traits examined showed significant phylogenetic signals according to both Blomberg's *K* and Pagel's λ values (Table 2). Among morphological traits, *K* values of all traits except RTD were significant (*P *<* *.05; Table 2). The highest *K* value belonged to LT (*K *=* *1.52), indicating strong phylogenetic signal; RD (*K *=* *0.62), SRL (*K *=* *0.48), SLA (*K *=* *0.46), and LTD (*K *=* *0.33) showed intermediate phylogenetic conservatism. Among chemical traits, only tissue N concentration displayed a significant phylogenetic signal (LN: *K *=* *0.33; RN: *K *=* *0.23; both *P *<* *.05). Similar results were given by Pagel's test.

**Table 2:** Phylogenetic signals (Blomberg's *K* and Pagel's λ) of leaf and absorptive root traits for different growth forms

	Trait	Woody species		Nonwoody species		All species	
		*K*	λ	*K*	λ	*K*	λ
Leaf	LT	**1.48**	**0.92**	0.29	0.00	**1.52**	**0.98**
	SLA	**0.52**	**0.56**	**0.36**	0.00	**0.46**	**0.85**
	LTD	**0.30**	**0.44**	0.30	0.14	**0.33**	**0.63**
	LC	0.27	**0.20**	**0.42**	0.99	0.19	**0.36**
	LN	**0.35**	**0.25**	0.24	0.04	**0.33**	**0.40**
Root	RD	**0.53**	**0.73**	**0.54**	**0.91**	**0.62**	**0.87**
	SRL	**0.34**	**0.38**	**0.47**	0.21	**0.48**	**0.56**
	RTD	0.19	**0.28**	**0.49**	**0.65**	0.19	**0.61**
	RC	0.17	0.00	0.25	0.03	0.15	0.00
	RN	**0.27**	**0.40**	**0.36**	0.27	**0.23**	**0.49**

Significance values are in bold (*P *<* *.05). Trait abbreviations are in Table 1.

Considerable differences in phylogenetic signals were found between woody and nonwoody species (Table 2). Among ten traits studied, seven traits of woody species showed significant phylogenetic signal according to Blomberg's *K*, as opposed to six traits of nonwoody species, but only two nonwoody traits were significant according to Pagel's λ (*P *<* *.05).

### 3.3 Trait relationships between leaves and absorptive roots

**Figure 1:**
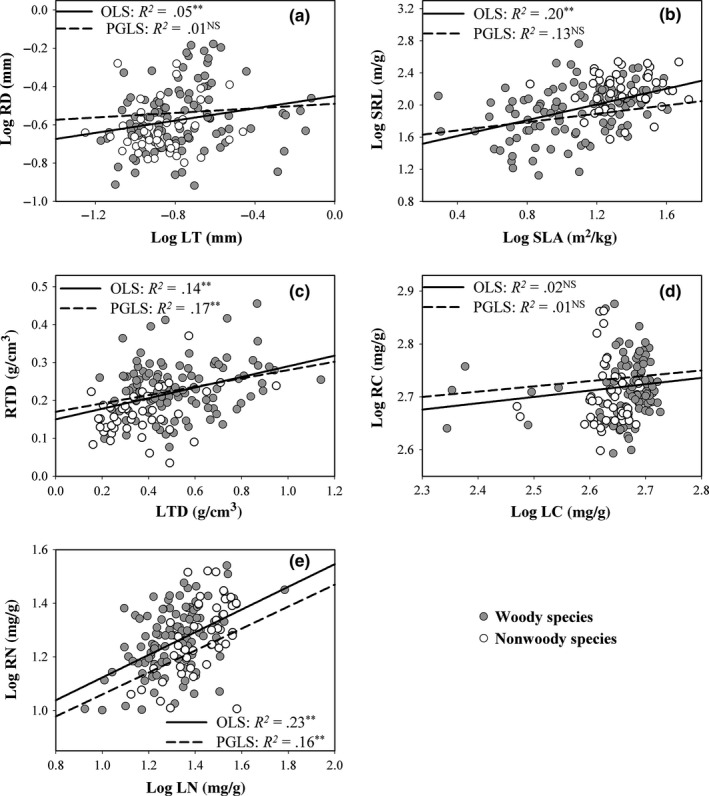
Relationships between leaf and absorptive root traits calculated using ordinary least squares (OLS) and phylogenetic generalized least squares (PGLS) methods across all species, woody, and nonwoody species, respectively. Model results of two methods are given in Table S3. Original data of tissue density are used here due to the problem with the scale of the response

Phylogenetic analysis suggested that there was a consistently positive relationship between leaf and absorptive root N concentrations, whereas their morphological trait correlations depended on whether phylogenetic information was considered and also differed between woody and nonwoody species (Figure 1 and Table S3). Generally, N concentrations of absorptive roots and leaves exhibited significantly positive association across all species and in both woody and nonwoody species datasets (all *P *<* *.001, Table S3). Moreover, the positive correlations of RN–LN were still retained even after adjusting for phylogenetic relatedness (Figure 1e and Table S3). However, among the morphological trait relationships, SRL–SLA relationships became insignificant after removing phylogenetic effect for woody species and all species (Table S3), and this correlation was not found in nonwoody plants (Figure 1b and Table S3). Similar results were found in the RD–LT relationship (Figure 1a and Table S3). But the positive correlation between RTD and LTD existed in both OLS and PGLS analyses and held for woody and total species (Figure 1c and Table S3). For RC–LC relationship, there were no significant correlations between above‐ and belowground organs under all conditions (*P *>* *.05, Figure 1d and Table S3).

We further conducted pPCA analysis on ten above and belowground traits using three different datasets, separately. Results showed that the first two axes of the pPCA explained 55.6–62.6% of overall variation (Table 3). The first pPCA axis explained 34.7% and 31.0% of the variance for woody and nonwoody plant species, respectively, and loaded most heavily on root morphological traits (e.g., SRL, RD, and RTD). The second pPCA factor described an additional 24.6% and 27.9% of the variance in these two datasets and loaded most heavily on leaf morphological traits, such as SLA and LTD. But when considering all species, the first pPCA axis was defined both root and leaf traits (i.e., SRL and SLA), and the second axis was driven mainly by root morphological traits (i.e., RD and SRL). Somewhat different from pPCA, the first axis of ordinary PCA was represented mainly by SRL and SLA, and the second axis loaded most heavily on SRL in these datasets of woody, nonwoody, and all species (Table S4).

**Table 3:** Loading scores of leaf and absorptive root traits on each component of the phylogenetic principal components analysis (pPCA)

	Woody species		Nonwoody species		All species	
	pPC1	pPC2	pPC1	pPC2	pPC1	pPC2
LT	0.58	0.52	0.47	0.14	−**0.62**	0.35
SLA	−0.54	−**0.81**	0.09	−**0.90**	**0.77**	−0.58
LTD	0.06	0.48	−0.51	**0.69**	−0.34	0.35
LC	0.19	0.29	−0.04	0.44	−0.26	0.23
LN	−0.35	−0.51	0.04	−0.54	0.52	−0.46
RD	**0.67**	−0.52	−0.37	−0.31	−0.48	−**0.69**
SRL	−**0.87**	0.47	**0.85**	0.33	**0.75**	**0.66**
RTD	0.24	0.11	−**0.64**	−0.07	−0.35	0.06
RC	0.05	0.00	0.26	−0.22	−0.04	0.05
RN	−0.37	−0.46	0.50	−0.40	0.50	−0.31
Variation explained (%)	34.7	27.9	31.0	24.6	36.6	25.0

Variable loading scores with the greatest load on each component are in bold. All the trait data are log_10_‐transformed prior to analysis. The abbreviations for the traits are in Table 1.

## 4 Discussion

### 4.1 Correlation in N concentration of leaves and absorptive roots

Significant correlation between leaf and root N concentrations has been well established in grasses and forbs (Craine et al., 2005; Kerkhoff et al., 2006; Tjoelker et al., 2005) and woody plants (Freschet, Bellingham, Lyver, Bonner, & Wardle, 2013; Valverde‐Barrantes et al., 2015). Similarly, our results indicated that N concentrations in leaves and absorptive roots were positively correlated, independent of phylogeny and plant growth form, which provides new support for the notion that N concentration could reflect inherent physiological and life‐history trade‐offs across the entire plant (Kerkhoff et al., 2006; Valverde‐Barrantes et al., 2015).

N is a fundamental component of all enzymes and proteins in plants; thus, N plays a critical role in organ function, growth rate, and plant life‐history strategies (Kerkhoff et al., 2006; Kong et al., 2016; Wright et al., 2004). In the process of plant growth, essential mineral nutrients are acquired from soils through roots, especially absorptive roots. Although plants obtain nutrients through multiple resource‐acquiring strategies of absorptive roots, these nutrients would be preferentially allocated to aboveground organ for leaf photosynthesis. Absorptive roots thus may act as the nutrient stock for aboveground leaves. Alternatively, high N content in roots may indicate the high capacity of resources acquirement and tissue metabolic activity, which is associated with increased photosynthetic export and phloem loading (Kerkhoff et al., 2006; Kong et al., 2016; Tjoelker et al., 2005). In this respect, N concentration could provide a valuable means for linking ecological perspectives on organisms and their environment from the whole‐plant level. In a recent analysis of multielement variability in different organs across Chinese forest biomes, Zhao et al. (2016) reported a coordinated pattern and similar elemental variability in the leaves and roots along the environmental gradients, implying that above‐ and belowground ecological processes pertaining to nutrient cycles are tightly linked.

Despite using standardized methods for leaves and absorptive root sampling, the correlation coefficients in leaf and root N concentrations remained lower than those reported in the leaf economics spectrum (Wright et al., 2004). One possible explanation is that the complexity of the soil environment, including the heterogeneity in soil nutrient availability and mycorrhizal fungi, presents a variety of constraints to root trait variation (Bardgett et al., 2014; Valverde‐Barrantes, Horning, Smemo, & Blackwood, 2016; Weemstra et al., 2016). In addition, the different above‐ and belowground physiological processes may weaken the relationship between leaf N and absorptive root N. In leaves, tissue N concentration is integral to the proteins of photosynthetic machinery, especially Rubisco. The strong correlations between leaf N and photosynthetic rate have been well‐verified across and within species at the global scale (Wright et al., 2004). But in roots, no single enzyme is mainly controlling resource acquisition (Chen et al., 2013), and whether the root uptake rates are as strongly related to root N content like in leaves remains controversial (Weemstra et al., 2016). Therefore, the above‐ and belowground correlation in a larger spectrum of plant traits, especially root function, is still needed to verify.

### 4.2 Decoupling between leaf and absorptive root morphology

In line with our expectation, morphological traits had decoupled patterns or weak linkages between above‐ and belowground organs after removing phylogenetic effect. Similar to our results, previous studies also pointed out that there are independent strategies between above‐ and belowground plant morphology (Craine et al., 2005; Geng et al., 2014; Tjoelker et al., 2005). Several hypotheses have been put forward to explain this phenomenon. First, the decoupled pattern may be a consequence of different selective pressures and constraints on morphological trait evolution between above‐ and belowground organs (Freschet et al., 2013; Kembel & Cahill, 2011; Mommer & Weemstra, 2012). Leaf traits are coordinated along a one‐dimensional axis, driven by maximizing light and CO_2_ capture while reducing resource loss by herbivores (Poorter et al., 2009). However, root traits are encountered by more complex abiotic and biotic selective pressures (Bardgett et al., 2014; Valverde‐Barrantes et al., 2016; Weemstra et al., 2016). Such constraints to root traits do not directly operate in leaf, resulting in a variety of belowground resource acquisition mechanisms and trade‐offs. Weemstra et al. (2016) recently proposed that, in contrast to a single acquisition–conservation axis in leaves, a multidimensional root trait framework may better accommodate and explain the variation in root traits observed across species.

Second, above‐ and belowground morphological traits may vary independently or even in opposite directions as a way to adapt to the multiple environmental gradients (Craine et al., 2005; Geng et al., 2014). Results of Geng et al. (2014) showed that SLA–SRL correlation shifted from positive to negative with the changes in the temperature of alpine grassland. Likely, different adaptive strategies in above‐ and belowground plant components may occur at temperate sites with both nutrient‐rich soil and cold temperature in our study (Table S1). For leaves, fast traits with high SLA and N concentration in photosynthetic tissues are advantageous in high‐resource environments (Poorter et al., 2009; Reich, 2014; Wright et al., 2004), whereas environmental extremes such as freezing soils might limit the exploration and prolongation of roots and select for low SRL and high RTD in roots. Therefore, different responses to the same growth environment may contribute to the weak coordination of morphology between above‐ and belowground organs.

A third potential explanation for the lack of clear integration between root and leaf morphology was advanced by Valverde‐Barrantes et al. (2015), who attributed this decoupled pattern to a stronger phylogenetic conservatism in root traits than in foliar tissues. This idea was only supported by nonwoody data in our study (Table 2). However, the significant relationships of RD–LT and SRL–SLA disappeared after removing phylogenetic relatedness (Figure 1a,b and Table S3), reflecting that these morphological linkages were controlled by phylogenetic affiliation. Indeed, compared with chemical traits, leaf and root morphological traits have been shown to be more phylogenetically conserved in this and previous studies (e.g., Kong et al., 2014; Li et al., 2015). Such strong phylogenetic effect acting on plant traits may explain why some trait correlations were strongly supported by early data collected from closely related species (e.g., Comas & Eissenstat, 2009; Fort, Jouany, & Cruz, 2013). Therefore, it is imperative to include phylogenetic considerations in the discussion of trait variation from the ecological to biogeographic scale and trait correlations at the whole‐plant level.

Finally, the morphological trait relationships between above‐ and belowground organs differed between woody and nonwoody species. There were significant SRL–SLA and RTD–LTD relationships for woody species but not for nonwoody species (Figure 1 and Table S3). Some previous studies also reported the different leaf–root trait correlations between woody and nonwoody species. For example, positive correlations between SLA and SRL have been found for woody species (Withington et al., 2006), but no significant relationships occurred when the species pools comprised woody and herbaceous species (Tjoelker et al., 2005) or only focused on herbaceous plants (Kembel & Cahill, 2011). The disparity of the trait relationship between woody and herbaceous plants may be attributable to difference in root branch system (Geng et al., 2014; Roumet et al., 2016). In woody species, a hierarchical root system is common and clear. It has been found that only the most distal one to three orders primarily serve resource acquisition functions (Guo et al., 2008; McCormack et al., 2015). However, the absorbing region of nonwoody species could be nearly as great as the extent of the root systems, which is largely different from the hierarchical branching architecture in woody plants. Meanwhile, for many herbaceous species, roots of small diameter may serve not only for mineral uptake, but also for anchorage and transport (Geng et al., 2014). Therefore, the first‐order root of herbs or grasses may not perform the same function as in woody species. Nevertheless, the methodology remains challenging for herbs that have very fine and breakable roots, and there is no consistent conclusion to define which orders in nonwoody roots are absorptive so far (Geng et al., 2014; Liu, He, Zeng, Lei, & Arndt, 2016; Roumet et al., 2016).

### 4.3 The controversy about above‐ and belowground trait relationships

Plant traits not only vary along environmental gradients but also show convergence in intra‐ and interspecific trait relationships across diverse taxonomic groups and biomes (Reich, 2014; Wright et al., 2004). The best known example is the leaf economics spectrum, which runs from species with rapid resource capture and a high relative growth rate, to those with contrasting traits commonly associated with conservative resource‐use strategies (Reich, 2014; Wright et al., 2004). However, the extent to which this paradigm can be extrapolated to other organs, such as roots and stems, and to the level of the whole plant remains controversial (Freschet et al., 2010, 2013; Mommer & Weemstra, 2012; Reich, 2014).

As both leaf economics and root traits are involved in the process of natural selection along trait trade‐off axes, the idea that economic trait spectra exist for different organs has been advanced (Freschet et al., 2010; Reich, 2014) and gained some support (e.g., Fort et al., 2013; Liu et al., 2010; Morales, Squeo, Tracol, Armas, & Gutierrez, 2015). On the other hand, evidence is accumulating in support of no ecological or complex linkages between leaves and root traits (Freschet et al., 2013; Valverde‐Barrantes et al., 2015; Weemstra et al., 2016), challenging a single spectrum of worldwide plant economics spectrum. For example, data from temperate grassland plant communities and tree species reported that there are complex or multidimensional relationships between corresponding leaf and root traits (Freschet et al., 2013; Kembel & Cahill, 2011). The complex soil environment as well as mycorrhizal interactions resulted in the functional difference between leaves and roots; thus, the resource economics syndromes that have been widely observed in leaves cannot be directly extrapolated to roots (Mommer & Weemstra, 2012; Weemstra et al., 2016).

In this study, our analyses of the above‐ and belowground trait relationships revealed that among traits related to plant resource uptake strategies, RN–LN was the only consistent root–leaf linkage, and such a correlation held for different growth forms and was not influenced by phylogenetic effect, whereas trait correlations in terms of morphology were not observed. These results suggest a complex integration of ecological linkages between above‐ and belowground resource acquisition organs. This is of paramount significance in understanding adaptive strategies of plants to highly heterogeneous environments and their influence on ecosystem process. First, the decoupling pattern in morphological traits allows for varieties of ecological strategies through adjusting independently above‐ and belowground morphologies to adapt to multiple environmental filters (Freschet et al., 2013; Laughlin, 2014). More combinations of trait dimensions may enable species to better adapt to multifarious niche dimensions, thus enhancing species coexistence and ecosystem stability (Laughlin, 2014). Second, owing to plant traits acting as drivers of many ecosystem processes and functions, the decoupled adjustments of leaf and root morphology along environmental gradients may lead to different life cycles of above‐ and belowground organs and plant–soil feedbacks (Bardgett et al., 2014; Westoby & Wright, 2006). Lastly, the generality of strong correlation in N concentration across plant organs and the fact that it applies across broad phylogenetic and biogeographic scales indicate that the general allocation rule governs the partitioning of nutrients above‐ and belowground, and thus have the potential to serve as a key trait in understanding the evolution of integrated suites of plant traits.

## 5 Conclusions

Using a large number of species from subtropical to temperate forests, our results showed that the relationships between above‐ and belowground traits might not be as simple as thought by plant economic spectrum theory. In this study, N concentrations of leaves and absorptive roots were highly coordinated, and the significantly positive RN–LN relationship was independent of both phylogenetic relatedness and growth forms. On the contrary, morphological traits of different plant organs were weakly related or decoupled after using phylogenetic analyses. Our results suggest the existence of a complex evolutionary strategy and multidimensional responses to environmental changes. This finding offers a new perspective for understanding the resource capture strategies of plants in adaptation to heterogeneous environments and contributes to the establishment of belowground trait datasets covering a wide range of species and biomes.

## Conflict of Interest

None declared.
