# Supplementary material for: Complex trait relationships between leaves and absorptive roots: Coordination in tissue N concentration but divergence in morphology
Source: Ecol Evol. 2017 Mar 19;7(8):2697–705. doi: 10.1002/ece3.2895 (PMC5395436; doi:10.1002/ece3.2895)
Supplement: Supplementary file 1 [file ECE3-7-2697-s001.docx]

**Complex trait relationships between leaves and absorptive roots: coordination in tissue N concentration but divergence in morphology**

Ruili Wang^1^, Qiufeng Wang^2^, Ning Zhao^3^, Guirui Yu^2^ & Nianpeng He^2^

^1^College of Forestry, Northwest A&F University, Yangling 712100, Shaanxi, China

^2^Synthesis Research Center of Chinese Ecosystem Research Network, Key Laboratory of Ecosystem Network Observation and Modeling, Institute of Geographic Sciences and Natural Resources Research, Chinese Academy of Sciences, Beijing 100101, China

^3^Laboratory of Remote Sensing and Geospatial Science, Cold and Arid Regions Environmental and Engineering Research Institute, Chinese Academy of Sciences, Lanzhou 730000, Gansu, China

**Running title:** Complexity of leaf and root trait relationships

**Correspondence**

Guirui Yu and Nianpeng He, Key Laboratory of Ecosystem Network Observation and Modeling, Institute of Geographic Sciences and Natural Resources Research, Chinese Academy of Sciences, Beijing 100101, China.

Tel: +86 10 64889432;

Fax: +86 10 64889432;

E-mail: [yugr@igsnrr.ac.cn](mailto:yugr@igsnrr.ac.cn) (G.Y) and henp@igsnrr.ac.cn (N.H.)

**Table S1.** Description of the selected sampling sites. DH, Dinghu Mountain; JL, Jiulian Mountain; SN, Shennongjia; TY, Taiyue Mountain; DL, Dongling Mountain; CB, Changbai Mountain; LS, Liangshui; HZ, Huzhong; MAT: mean annual temperature; MAP: mean annual precipitation; Soil N, soil total nitrogen concentration; Soil P, soil total phosphorus concentration.

| Site | Latitude  (°N) | Longitude  (°E) | Altitude  (m) | MAT  (°C) | MAP (mm) | Soil N  (mg g^-1^) | Soil P  (mg kg^-1^) | Soil type | Vegetation type |
| --- | --- | --- | --- | --- | --- | --- | --- | --- | --- |
| DH | 23.17 | 112.54 | 240 | 20.9 | 1927.0 | 1.76 | 203.68 | Lateritic red soil | Subtropical evergreen broadleaved forest |
| JL | 24.58 | 114.44 | 562 | 16.7 | 1954.0 | 2.35 | 358.78 | Red soil | Subtropical evergreen broadleaved forest |
| SN | 31.32 | 110.50 | 1510 | 10.6 | 1330.0 | 3.76 | 832.50 | Yellow brown soil | Subtropical mixed evergreen and deciduous broadleaved forest |
| TY | 36.70 | 112.08 | 1668 | 6.2 | 662.0 | 2.56 | 522.75 | Cinnamon soil | Temperate deciduous broadleaved forest |
| DL | 39.96 | 115.42 | 972 | 4.8 | 539.1 | 3.12 | 556.38 | Brown soil | Temperate deciduous broadleaved forest |
| CB | 42.40 | 128.09 | 758 | 2.6 | 691.0 | 6.37 | 1797.88 | Dark brown soil | Temperate mixed forest |
| LS | 47.19 | 128.90 | 401 | -0.3 | 676.0 | 4.59 | 565.20 | Dark brown soil | Temperate mixed forest |
| HZ | 51.78 | 123.02 | 850 | -4.4 | 481.6 | 3.15 | 815.80 | Grey forest soil | Cold-temperate coniferous forest |

**Table S2.** Akaike’s information criterion (AIC) values for trait relationships between leaf and absorptive root with different phylogenetic correlation structures and ordinary least squares (OLS). Models with the lowest AIC values are selected as the best phylogenetic correlation structure for phylogenetic generalized least squares (PGLS) analyses; the best models are in bold.

|  |  | Brownian | Martin’s | Pagel’s | OLS |
| --- | --- | --- | --- | --- | --- |
| RD-LT | Woody species | -96.08 | -86.81 | **-117.73** | -88.81 |
|  | Non-woody species | -44.39 | -42.74 | **-79.95** | -44.74 |
|  | All species | -149.88 | -133.95 | **-175.34** | -135.95 |
| SRL-SLA | Woody species | 42.7 | 46.42 | **24.43** | 44.42 |
|  | Non-woody species | 8.52 | 5.76 | **4.34** | 3.76 |
|  | All species | 38.42 | 54.58 | **27.44** | 52.58 |
| RTD-LTD | Woody species | -62.41 | -129.45 | **-289.81** | -131.45 |
|  | Non-woody species | -11.23 | -19.58 | **-20.45** | -21.57 |
|  | All species | -82.64 | -131.87 | **-402.82** | -133.87 |
| RC-LC | Woody species | -235.86 | -334.9 | **-356.72** | -336.9 |
|  | Non-woody species | -88.52 | -108.27 | **-108.33** | -110.27 |
|  | All species | -347.74 | -448.93 | **-465.55** | -450.93 |
| RN-LN | Woody species | -140.59 | -183.64 | **-188.84** | -185.64 |
|  | Non-woody species | -56.22 | -55.57 | **-57.57** | -57.56 |
|  | All species | -206.97 | -239.42 | **-257.53** | -241.42 |

Brownian, Brownian correlation structure; Martin’s, Martin’s correlation structure; Pagel’s, Pagel’s correlation structure.

Trait abbreviation: LT, leaf thickness; SLA, specific leaf area; LTD, leaf tissue density; LC, leaf carbon concentration; LN, leaf nitrogen concentration; RD, root diameter; SRL, specific root length; RTD, root tissue density; RC, root carbon concentration; RN, root nitrogen concentration.

**Table S3.** Model outputs for ordinary least squares (OLS) and phylogenetic generalized least squares (PGLS) regressions of the leaf-root trait relationships in the dataset of woody, non-woody and all species, respectively. Abbreviations: λ, Pagel’s lambda; Log-lik, log likelihood. The trait abbreviations are given in Table S2. Statistically significant relationships are in bold (*P* < 0.05). Except RTD and LTD, all trait data were log_10_-transformed prior to analysis.

| Woody species | | Slope | Intercept | *R^2^* | *P* | λ | Log-Lik |
| --- | --- | --- | --- | --- | --- | --- | --- |
| RD-LT | OLS | 0.16 | -0.45 | 0.04 | **0.027** |  |  |
|  | PGLS | 0.05 | -0.44 |  | 0.491 | 0.68 | 62.86 |
| SRL-SLA | OLS | 0.44 | 1.44 | 0.16 | **< 0.001** |  |  |
|  | PGLS | 0.20 | 1.54 | 0.08 | 0.075 | 0.69 | -8.21 |
| RTD-LTD | OLS | 0.18 | -0.60 | 0.04 | **0.027** |  |  |
|  | PGLS | 0.08 | 0.20 | 0.02 | **0.016** | 0.34 | 148.91 |
| RC-LC | OLS | 0.11 | 2.42 | 0.02 | 0.169 |  |  |
|  | PGLS | 0.09 | 2.49 | 0.06 | 0.221 | 0.11 | 182.36 |
| RN-LN | OLS | 0.43 | 0.71 | 0.24 | **< 0.001** |  |  |
|  | PGLS | 0.39 | 0.75 | 0.28 | **< 0.001** | 0.37 | 98.42 |

| Non-woody species | | Slope | Intercept | *R^2^* | *P* | λ | Log-Lik |
| --- | --- | --- | --- | --- | --- | --- | --- |
| RD-LT | OLS | 0.10 | -0.53 | 0.02 | 0.437 |  |  |
|  | PGLS | 0.03 | 0.29 | 0.003 | 0.881 | 0.91 | 43.98 |
| SRL-SLA | OLS | 0.11 | 2.03 | 0.01 | 0.592 |  |  |
|  | PGLS | -0.02 | 2.11 | 0.01 | 0.918 | 0.37 | 1.83 |
| RTD-LTD | OLS | 0.22 | -0.71 | 0.05 | 0.159 |  |  |
|  | PGLS | 0.14 | -0.72 | 0.03 | 0.345 | 0.39 | 14.23 |
| RC-LC | OLS | 0.04 | 2.60 | 0.001 | 0.861 |  |  |
|  | PGLS | 0.02 | 2.64 | < 0.001 | 0.926 | 0.03 | 58.17 |
| RN-LN | OLS | 0.65 | 0.35 | 0.30 | **< 0.001** |  |  |
|  | PGLS | 0.70 | 0.24 | 0.25 | **< 0.001** | 0.15 | 32.11 |

| All species | | Slope | Intercept | *R^2^* | *P* | λ | Log-Lik |
| --- | --- | --- | --- | --- | --- | --- | --- |
| RD-LT | OLS | 0.16 | -0.45 | 0.05 | **0.008** |  |  |
|  | PGLS | 0.06 | -0.49 | 0.01 | 0.325 | 0.78 | 91.67 |
| SRL-SLA | OLS | 0.49 | 1.42 | 0.20 | **< 0.001** |  |  |
|  | PGLS | 0.26 | 1.58 | 0.13 | 0.055 | 0.79 | -9.72 |
| RTD-LTD | OLS | 0.14 | 0.15 | 0.14 | **< 0.001** |  |  |
|  | PGLS | 0.11 | 0.17 | 0.17 | **< 0.001** | 0.53 | 205.41 |
| RC-LC | OLS | 0.12 | 2.40 | 0.02 | 0.119 |  |  |
|  | PGLS | 0.10 | 2.47 | 0.01 | 0.130 | -0.05 | 236.77 |
| RN-LN | OLS | 0.42 | 0.70 | 0.23 | **< 0.001** |  |  |
|  | PGLS | 0.41 | 0.65 | 0.16 | **< 0.001** | 0.60 | 132.77 |

**Table S4.** Loading scores of leaf and absorptive root traits on each component of the conventionally principal components analysis (PCA). Variable loading scores with the greatest loading on each component appear in bold. All the trait data were log_10_-transformed prior to analysis. The abbreviations for the traits are given in Table S2.

|  | Woody speceis | |  | Non-woody species | |  | All species | |
| --- | --- | --- | --- | --- | --- | --- | --- | --- |
|  | PC1 | PC2 |  | PC1 | PC2 |  | PC1 | PC2 |
| LT | 0.41 | 0.29 |  | -0.11 | 0.04 |  | 0.34 | 0.27 |
| SLA | **-0.58** | -0.44 |  | -0.26 | 0.49 |  | **-0.59** | -0.45 |
| LTD | 0.16 | 0.16 |  | 0.38 | **-0.53** |  | 0.23 | 0.19 |
| LC | 0.06 | 0.03 |  | 0.01 | -0.06 |  | 0.06 | 0.04 |
| LN | -0.21 | -0.20 |  | -0.12 | 0.23 |  | -0.21 | -0.20 |
| RD | 0.24 | -0.41 |  | 0.26 | 0.34 |  | 0.21 | -0.41 |
| SRL | **-0.59** | **0.65** |  | **-0.74** | **-0.50** |  | **-0.60** | **0.66** |
| RTD | 0.10 | 0.18 |  | 0.25 | -0.13 |  | 0.17 | 0.16 |
| RC | 0.02 | 0.01 |  | -0.09 | 0.04 |  | 0.01 | 0.01 |
| RN | -0.09 | -0.19 |  | -0.29 | 0.20 |  | -0.09 | -0.16 |
| Variation  explained (%) | 53.2 | 25.6 |  | 33.5 | 22.6 |  | 52.4 | 22.1 |

**Figure S1.** Phylogenetic tree of 154 species sampled in this study. Scale bar = 100 Ma (million years ago).

**
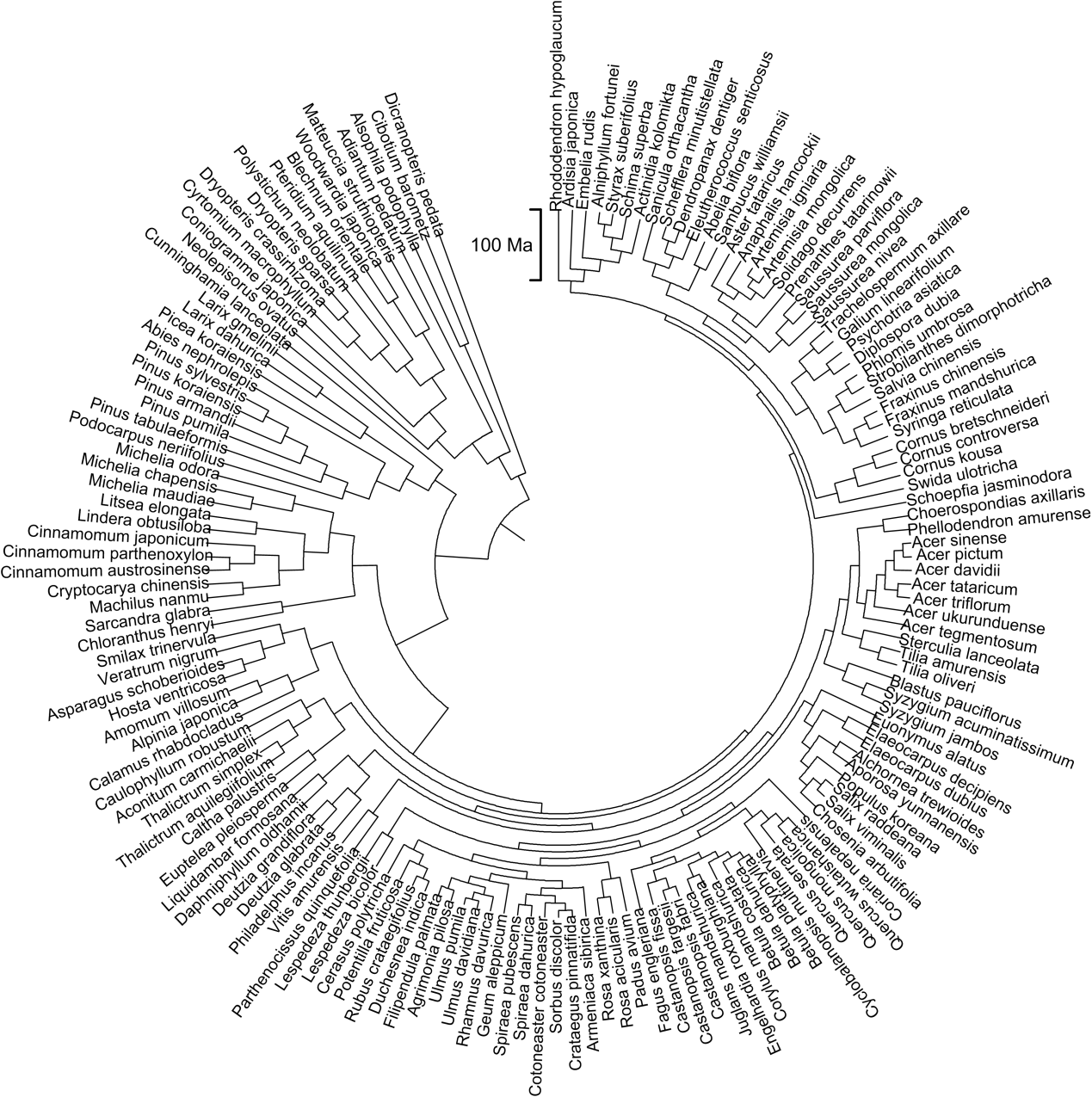
**

**Figure S2.** Histograms showing the distributions of ten leaf and absorptive root traits for all 154 species. Bold curves indicate fitted log-normal curves. Min, mimimum value; Max, maximum; CV, coefficient of variation. Trait abbreviations are provided in Table S2.

**

**
